# Supplementary material for: The RALE Score Versus the CT Severity Score in Invasively Ventilated COVID-19 Patients—A Retrospective Study Comparing Their Prognostic Capacities
Source: Diagnostics (Basel). 2022 Aug 26;12(9):2072. doi: 10.3390/diagnostics12092072 (PMC9497927; doi:10.3390/diagnostics12092072)
Supplement: Supplementary file 1 [file diagnostics-12-02072-s001.zip › diagnostics-1867537-supplementary-done.pdf]

diagnostics

# Supplementary material

# The RALE score versus the CT Severity Score in Invasively Ventilated COVID–19 Patients—A Retrospective Study Comparing Their Prognostic Capacities

**Figure S1.** The RALE scoring sheet, showing the total score and the score for each of the four quadrants for a representative study patient.

RALE, radiographic assessment of lung edema; Q, chest x-ray quadrant.

**Consolidation[a]**

| Consolidation Score | Extent of alveolar opacities |
|---|---|
| 0 | None |
| 1 | < 25 % |
| 2 | 25 – 50 % |
| 3 | 50 – 75 % |
| 4 | > 75 % |

**Calculation of the RALE score for radiograph**

| Score | Q1 | Q2 | Q3 | Q4 | Total |
|---|---|---|---|---|---|
| Consolidation | 2 | 1 | 3 | 4 | |
| Density | 3 | 3 | 3 | 3 | |
| Quadrant Score | 2 x 3 = 6 | 1 x 3 = 3 | 3 x 3 = 9 | 4 x 3 = 12 | 30 |

**Density[b]**

| Density Score | Density of alveolar opacities |
|---|---|
| 1 | Hazy |
| 2 | Moderate |
| 3 | Dense |

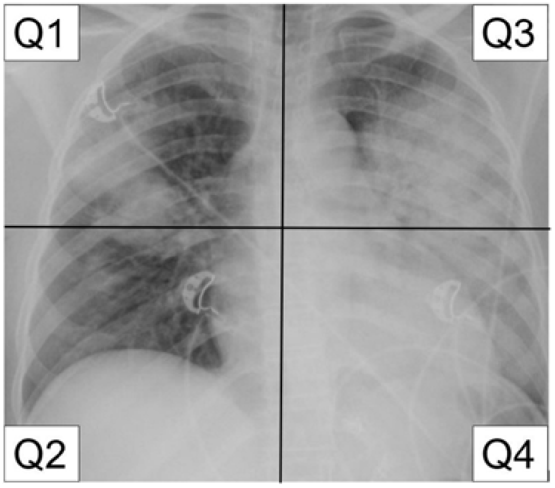


**Final RALE Score[c]**

| Right Lung | Left Quadrant |
|---|---|
| Upper Quadrant<br>Cons x Den = Q1 Score | Upper Quadrant<br>Cons x Den = Q3 Score |
| Lower Quadrant<br>Cons x Den = Q2 Score | Lower Quadrant<br>Cons x Den = Q4 Score |

**Total RALE = Q1 + Q2 + Q3 + Q4**

[a] Consolidation is scored for each quadrant
[b] Density is scored for each quadrant having a consolidation > 0
[c] If Quadrant consolidation Score is 0 then Quadrant score is 0

**Table S1.** The CT severity score per lobe. A system for scoring ground-glass opacity, interstitial opacity, and air trapping on thin-section CT scan.

| **Score** | **Definition** |
|---|---|
| **0** | None |
| **1** | <5% of lobe* |
| **2** | 5%–25% of lobe |
| **3** | 26%–49% of lobe |
| **4** | 50%–75% of lobe |
| **5** | >75% of lobe |
| *minimal but not normal<br><br>**Maximum of 25 point** | |

A

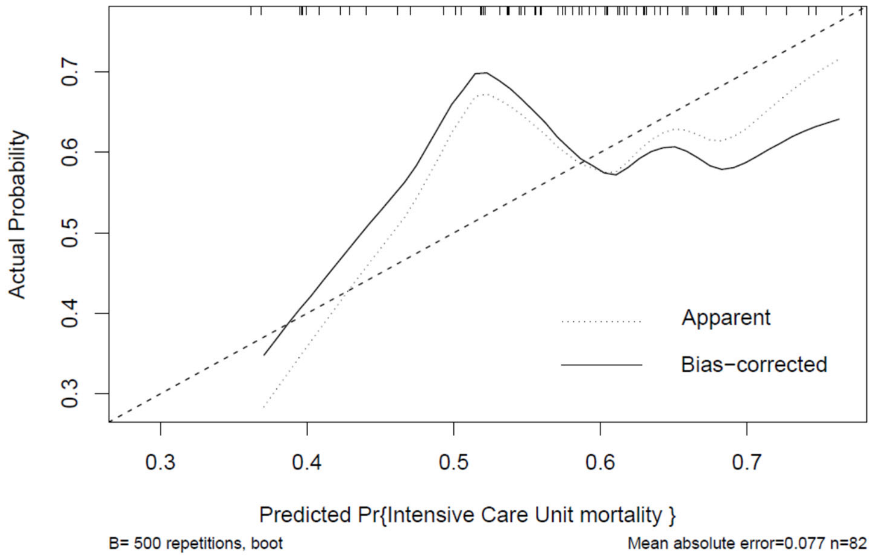

B

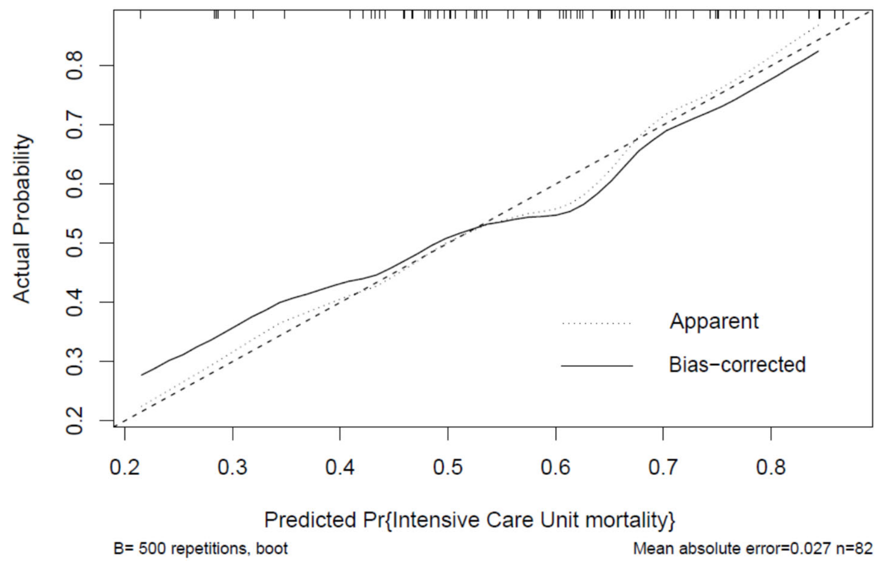


**Figure S2.** Calibration plots of the fitted model of (A) the RALE score and (B) the CTSS.

A

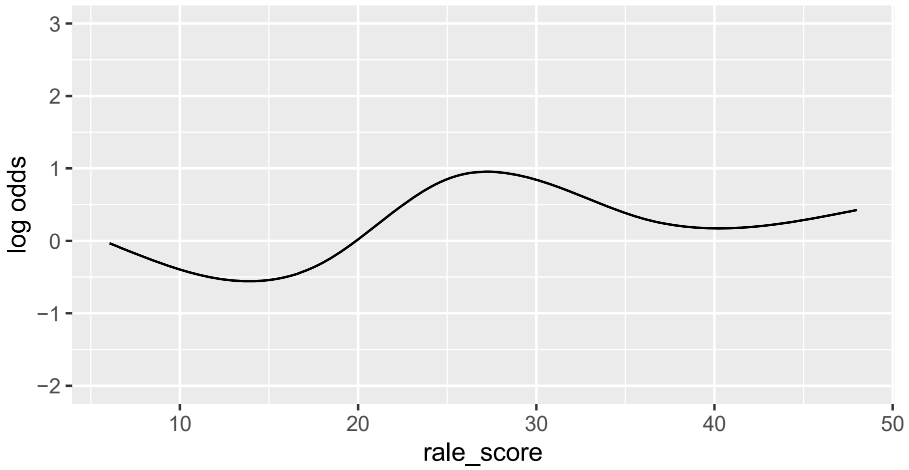

B

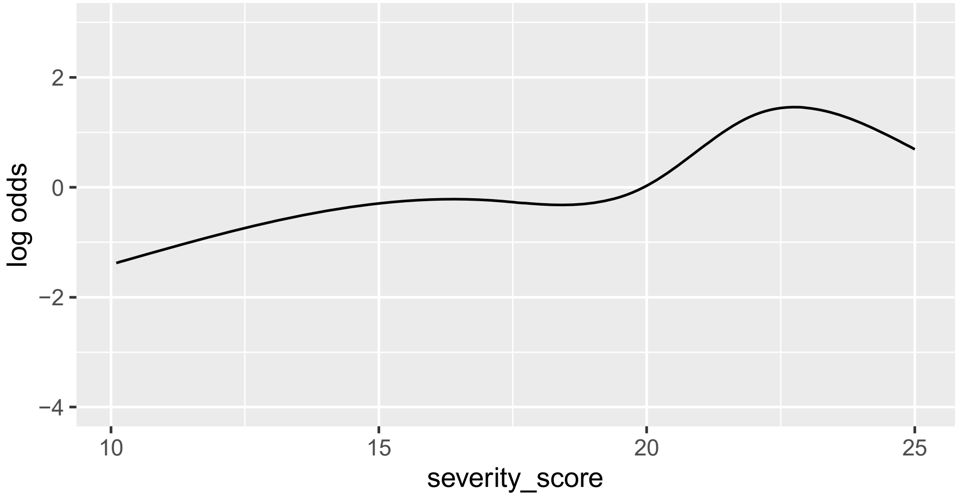


**Figure S3.** Spline graphical association of the RALE score (panel A) and CTSS (panel B) versus ICU mortality, adjusted median APACHE II of 12 score and BMI of 29.
